# Contributions of the Nucleus Accumbens Shell in Mediating the Enhancement in Memory Following Noradrenergic Activation of Either the Amygdala or Hippocampus

**DOI:** 10.3389/fphar.2018.00047

**Published:** 2018-02-02

**Authors:** Erin C. Kerfoot, Cedric L. Williams

**Affiliations:** Division of Neuroscience and Behavior, Department of Psychology, University of Virginia, Charlottesville, VA, United States

**Keywords:** emotions, memory, nucleus accumbens, norepinephrine, amygdala and hippocampus, inhibitory avoidance, memory modulation

## Abstract

The nucleus accumbens shell is a site of converging inputs during memory processing for emotional events. The accumbens receives input from the nucleus of the solitary tract (NTS) regarding changes in peripheral autonomic functioning following emotional arousal. The shell also receives input from the amygdala and hippocampus regarding affective and contextual attributes of new learning experiences. The successful encoding of affect or context is facilitated by activating noradrenergic systems in either the amygdala or hippocampus. Recent findings indicate that memory enhancement produced by activating NTS neurons, is attenuated by suppressing accumbens functioning after learning. This finding illustrates the significance of the shell in integrating information from the periphery to modulate memory for arousing events. However, it is not known if the accumbens shell plays an equally important role in consolidating information that is initially processed in the amygdala and hippocampus. The present study determined if the convergence of inputs from these limbic regions within the nucleus accumbens contributes to successful encoding of emotional events into memory. Male Sprague-Dawley rats received bilateral cannula implants 2 mm above the accumbens shell and a second bilateral implant 2 mm above either the amygdala or hippocampus. The subjects were trained for 6 days to drink from a water spout. On day 7, a 0.35 mA footshock was initiated as the rat approached the spout and was terminated once the rat escaped into a white compartment. Subjects were then given intra-amygdala or hippocampal infusions of PBS or a dose of norepinephrine (0.2 μg) previously shown to enhance memory. Later, all subjects were given intra-accumbens infusion of muscimol to functionally inactivate the shell. Muscimol inactivation of the accumbens shell was delayed to allow sufficient time for norepinephrine to activate intracellular cascades that lead to long-term synaptic modifications involved in forming new memories. Results show that memory improvement produced by infusing norepinephrine in either the amygdala or hippocampus is attenuated by interrupting neuronal activity in the shell 1 or 7 7 h following amygdala or hippocampus activation. These findings suggest that the accumbens shell plays an integral role modulating information initially processed by the amygdala and hippocampus following exposure to emotionally arousing events. Additionally, results demonstrate that the accumbens is involved in the long-term consolidation processes lasting over 7 h.

## Introduction

In response to new learning episodes, the shell division of the accumbens receives a constellation of neural input from brain regions that encode separate attributes of novel experiences. These inputs include signals from brainstem nuclei representing physiological changes induced by the event, information from the amygdala regarding the affective appraisal of stimuli and representations of contextual and temporal relationships of the episode from the ventral hippocampus (Mogenson et al., 1980; Groenewegen et al., 1987; Meredith et al., 1990; Wang et al., 1992; Brog et al., 1993; Petrovich et al., 1996; Delfs et al., 1998; Al’bertin, 2003; French and Totterdell, 2003; Jongen-Relo et al., 2003; McGinty and Grace, 2009). Interestingly, projections from the basolateral amygdala (BLA) and ventral hippocampus (HIPP) converge monosynaptically on projection neurons within the accumbens shell (French and Totterdell, 2003; Stuber et al., 2011; Britt et al., 2012). Neurons in the shell are depolarized following either high frequency or optogenetic stimulation of the BLA and these effects are suppressed by inactivating the HIPP (Gill and Grace, 2011; Correia et al., 2016). Based on this anatomical arrangement, it is suggested that information transmitted from either the amygdala or hippocampus in response to new learning may require extensive processing within the nucleus accumbens (Roozendaal et al., 2001; Correia et al., 2016). The accumbens shell therefore, may be in a critical position to modulate information emanating from limbic structures that encode separate features of newly experienced events.

Interactions between the basolateral amygdala, hippocampus and accumbens shell may involve unique roles in encoding the motivational and affective value of stimuli as well as contextual representations during new learning. Disrupting connections between the BLA and accumbens impairs the capacity of laboratory animals to acquire second-order conditioned responses (Setlow et al., 2002). Although rats with unilateral basolateral and contralateral accumbens lesions show no deficits in learning that a light stimulus signals food availability, the lesioned animals fail to acquire more complex associations such as learning that a tone presented before the light also signals food availability. Additionally, previous studies revealed that ventral hippocampus lesions impair memory for contextual fear conditioning, whereas dorsal lesions disrupt spatial learning in tasks such as the Morris water maze task (Richmond et al., 1999; Burhans and Gabriel, 2007; Ji and Maren, 2008; Czerniawski et al., 2009). Given that the ventral hippocampus projects to the accumbens shell (Groenewegen et al., 1987), it is not surprising that animals with accumbens shell lesions show reduced freezing and by implication, poorer retention when returned to the training context previously paired with an aversive footshock during initial conditioning (Jongen-Relo et al., 2003). Together, these studies suggest a functional relationship between the amygdala, ventral hippocampus and accumbens shell.

In light of the collective behavioral, neurochemical and anatomical evidence, Experiment 1 addressed whether memory enhancement produced by activating the BLA or HIPP following an emotionally arousing learning experience requires neuronal processing within the accumbens shell. In the present study, a surprising footshock was administered during training and followed by an intra-amygdala or hippocampal infusion of norepinephrine. If the accumbens shell contributes to this process, it is important to identify the timeframe that information initially encoded by the amygdala and hippocampus is further modified and processed within the accumbens shell. To address this issue, subjects received intra-amygdala or hippocampal infusion of norepinephrine at a dose previously shown to enhance memory (Hatfield and McGaugh, 1999; LaLumiere et al., 2003) and then given intra-accumbens infusion of a PBS control solution or muscimol to functionally inactivate the shell. Muscimol inactivation of the accumbens shell was delayed for 1 or 7 h to allow sufficient time for norepinephrine to activate intracellular cascades that lead to long-term synaptic modifications involved in forming new memories (Bergado et al., 2007; Alberini, 2009; Inda et al., 2011). If the accumbens mediates the consequences of limbic activation, then inactivation of the shell should attenuate memory for the aversive experience despite noradrenergic activation of either the hippocampus or amygdala.

In Experiment 2, a subset of the animals trained in the first study were exposed 48 h later to a generalization test in a Y-maze apparatus. The left and right allies were designed to have either similar or completely different contextual features as the original training with footshock in Experiment 1, and the latency to avoid the context with similar features was recorded. Findings from this study were expected to reveal if long term representations of the training context were differentially strengthened in the hippocampus relative to the amygdala by posttraining infusions of norepinephrine into each structure following footshock delivery in Experiment 1. If memories are strengthened by activating these limbic structures, then certain aspects of the memory trace should be evident when the same animals are given a generalization-test in a new apparatus constructed with similar and different contextual features as the original training environment.

## General Methods

All experimental procedures were performed in accordance with the NIH Guide for Care and Use of Laboratory Animals and approved by the University of Virginia’s Institutional Animal Care and Use Committee.

### Subjects

Seventy-four male Sprague-Dawley rats (275–300 g) obtained from Charles River Laboratories (Wilmington, MA, United States) were used in these experiments. The rats were individually housed in polypropylene cages with corncob bedding and maintained on a standard 12:12 h light-dark cycle with lights on at 7:00 A.M. Food and water were available ad libitum during the 7 days adaptation period to the vivarium.

### Surgery

Each rat received an injection of atropine sulfate (0.1 mg/kg i.p., American Pharmaceutical Partners, Inc., Schaumburg, IL, United States) and anesthetized with sodium pentobarbital (50 mg/kg, i.p., Abbot Laboratories, North Chicago, IL, United States). A midline scalp incision was made and 15 mm long extra thin wall stainless steel guide cannula (25 gauge, Small Parts, Miami Lakes, FL, United States) were implanted bilaterally in all subjects, 2 mm above the nucleus accumbens shell (AP +0.7, ML ± 1.0 from bregma, DV -5.4 from skull surface). Separate groups received additional bilateral cannula implants 2 mm above either the basolateral amygdala (AP -3.0, ML ± 5.0 from bregma, DV -6.7 from skull surface; *n* = 36) or the ventral subiculum of the hippocampus (AP -5.3, ML ± 4.5, DV -8.6 from skull surface; *n* = 34). All coordinates were adapted from the atlas of Paxinos and Watson (2005). Guide cannulae and two skull screws for anchoring were affixed to the skull with dental cement. The scalp was closed with sutures and stylets (15 mm, 00 insect dissection pins) were inserted into the four, implanted injection cannulae to prevent occlusion. Penicillin (0.1 ml i.m., Fort Dodge Animal Health, Fort Dodge, IA, United States) was administered immediately after surgery along with the analgesic, buprenex (0.05 ml s.c., Hospira, Inc., Lake Forrest, IL, United States). The rats remained in a temperature-controlled chamber for at least 1-h following surgery and were given 7 days to recover before initiating water deprivation procedures and behavioral training.

### Histology

**FIGURE 1:**
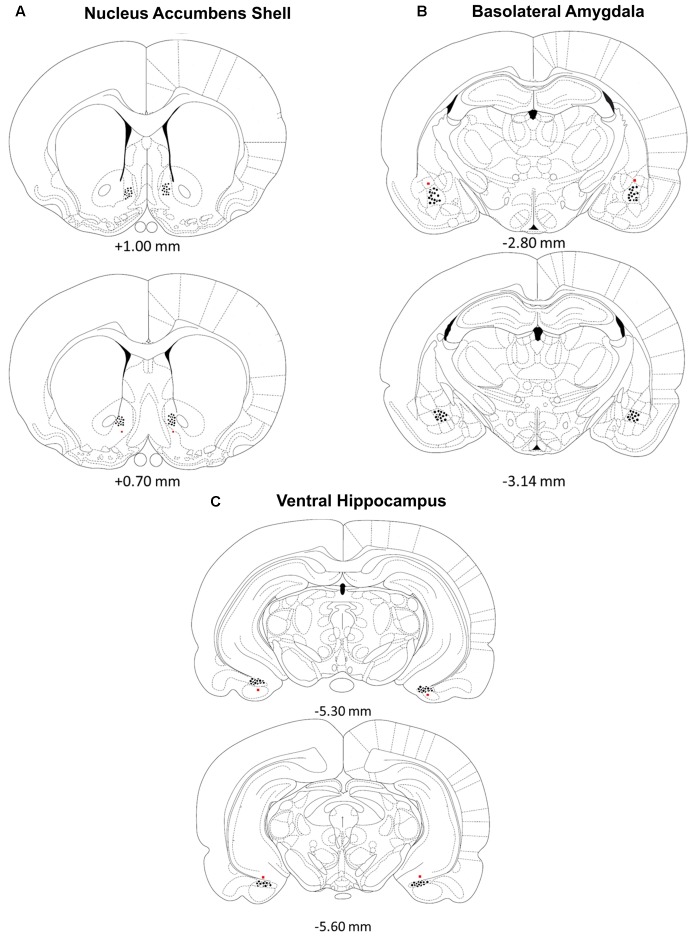
**(A–C)** Location of injection needle tip placements in the **(A)** nucleus accumbens shell, **(B)** basolateral amygdala and **(C)** ventral hippocampus. Brain diagram from “The Rat Brain in Stereotaxic Coordinates”; adapted from Paxinos and Watson, 2005.

Rats were deeply anesthetized with a euthanasia solution and perfused intracardially with 0.9% saline followed by 10% formalin to verify microinjection cannulae placement. The brains were stored in a 10% formalin and 12% sucrose solution until sectioned on a vibratome. Sections were cut 60 μm thick, mounted on glass slides, subbed with chromium-aluminum and stained with cresyl violet. The location of the cannulae and injection needle tips were verified by examining enlarged projections of the slides. Data from animals with incorrect placements of guide cannula extending beyond the boundaries of the accumbens shell, basolateral amygdala complex or ventral hippocampus were excluded from statistical analysis. These criteria resulted in excluding the data of four animals from the BLA /Norepinephrine NAC-1hr muscimol, BLA/PBS NAC-1hr muscimol, HIPP /Norepinephrine NAC-1hr MUSC, and HIPP/PBS NAC-1hr PBS groups. A composite illustrating the location of injection needle tips within each of the three brain regions is shown in **Figure 1**.

## Methods for Experiment 1

### Behavioral Paradigm: Water-Motivated Inhibitory Avoidance Task

#### Apparatus

A trough-shaped, two compartment rectangular apparatus (91 cm long, 21 cm wide at the top and 6.4 cm wide at the bottom) with a hinged lid was used to train the rats in a water-motivated inhibitory avoidance task. A sliding metal door (14.5 cm) separated a white and dark compartment. The white compartment was constructed of white opaque Plexiglas (31 cm long) and brightly illuminated by a 60 watt light located directly above the compartment. The dark compartment was constructed of stainless steel plates (60 cm long). A curved stainless steel water spout connected to a 30-cc plastic syringe containing water was placed 1 cm above the floor at the end of the dark compartment.

#### Training

One-week after surgery, rats were placed on a water restriction schedule with access to water for 20 min a day in addition to water consumed during behavioral training. Body weights were monitored daily to ensure weights did not deviate below 10% of their ad-lib feeding weights throughout the experiment. Animals were habituated by placing each in the inhibitory avoidance apparatus for 300 s to cross between the white and dark compartment and to explore the drinking spout.

For the next 6 days of training each rat was placed in the dark compartment facing the retractable door that separated the dark from the illuminated compartment. The metal door was lowered to 2/3 of its length (i.e., creating a 4 cm hurdle). A timer was started and the following measures were recorded until the completion of the trial: (a) latency to begin drinking, (b) total amount of time spent drinking, (c) total amount of time spent in the dark compartment and (d) total amount of time spent in the white illuminated compartment. Each training day consisted of one trial lasting 120 s.

On Day 7 (i.e., *experimental day*), each rat was placed in the dark compartment as before however, a 0.35 mA electrical footshock was administered manually as the rat initiated a lick toward the water spout. The shock remained on until the animal escaped the dark compartment by jumping over the 4 cm high hurdle into the illuminated white compartment. Each animal was retained in the white compartment for 30 s with the door two-thirds open. The door was then raised and animals were retained in the white compartment for an additional 30 s. Hence, the animals were allowed 60 s to learn that the white illuminated compartment was safe relative to the dark compartment where footshock was just experienced. Each animal was then removed from the apparatus and given intra-amygdala or hippocampal infusions of PBS or a dose of norepinephrine (0.2 μg/0.5 μl) previously shown to enhance memory (Izquierdo et al., 1992; Hatfield and McGaugh, 1999; LaLumiere et al., 2003). At either 1 or 7 h after the shock and amygdala or hippocampus injection, all subjects were removed from their home cages and given an intra-accumbens infusion of either muscimol (100 ng / 0.5 μl) or PBS. The dose of muscimol was based upon those that have been shown to impair memory in the accumbens shell (Scheel-Kruger et al., 1977; Reynolds and Berridge, 2002; Yang et al., 2005).

#### Microinjection Procedure

Each experimental rat was restrained by hand in the experimenter’s lap, the stylets were removed and 17 mm, 30 gauge injection needles were inserted bilaterally into either the basolateral amygdala or ventral hippocampus. These injections were followed 1 or 7 h later by bilateral injections of PBS or muscimol (100 ng/0.5 μl) into the accumbens shell. The tip of the injection needles extended 2 mm beyond the base of the guide cannulae. The needles were connected to 10 μl Hamilton syringes by PE-20 (polyethylene) tubing. An automated syringe pump (Sage-Orion, Boston, MA, United States) delivered 0.5 μl PBS, norepinephrine (0.2 μg; Sigma–Aldrich, St. Louis, MO, United States) or muscimol (100 ng; Sigma–Aldrich, St. Louis, MO, United States) over 60 s. The injection needles were left in place for an additional 60 s following infusions to ensure complete delivery of the drugs and the stylets were then reinserted into the cannulae. The number of subjects randomly assigned to the six basolateral amygdala (BLA) treatment groups is included in parenthesis *(1) BLA /PBS-NAC PBS 1hr = 5; (2) BLA /Noreipnephrine-NAC PBS 1 h = 7; (3) BLA /PBS-NAC Muscimol 1 h = 5; (4) BLA /Noreipnephrine-NAC Muscimol 1 h = 5; (5) BLA /PBS-NAC Muscimol 7 h = 5; (6) BLA /Noreipnephrine-NAC Muscimol 7 h = 5.*

The following subjects were included in the separate ventral hippocampus (HIPP) groups: *(1) HIPP/PBS-NAC PBS 1 h = 5; (2) HIPP /Noreipnephrine-NAC PBS 1 h = 5; (3) HIPP /PBS-NAC Muscimol 1 h = 5; (4) HIPP /Noreipnephrine-NAC Muscimol 1 h = 7; (5) HIPP /PBS-NAC Muscimol 7 h = 6; (6) HIPP/Noreipnephrine-NAC Muscimol 7 h = 6.*

#### Retention Test

Memory for the surprising footshock in the dark compartment was assessed 24 h later and consisted of two phases. During Phase 1, the rats were placed in the dark compartment facing the partially lowered metal door and given 60 s to enter the white compartment or alternatively, to initiate the first lick from the water spout. If the rat entered the white compartment, the metal door was raised and the rat remained in the white compartment for 30 s. Phase 2 of retention began after this 30-s period. Those that did not enter the white compartment after 60 s were removed and placed in the white compartment with the metal door raised for 30 s. Measures recorded during Phase 1 included latency to drink, amount of time spent drinking, and latency to escape into the white compartment. During Phase 2, the metal door was lowered and the time spent avoiding the dark compartment, latency to drink from the spout and total time spent drinking was recorded over 300 s.

#### Statistical Analysis

The behavioral measures from the water-motivated inhibitory avoidance task are expressed as mean ± standard errors (SE). Between-group comparisons for the behaviors measured during retention testing were made with a 2 × 3 analysis of variance (ANOVA) followed by *post hoc* tests.

## Methods for Experiment 2

### Behavioral Paradigm: Y-Maze Task

#### Apparatus

**FIGURE 2:**
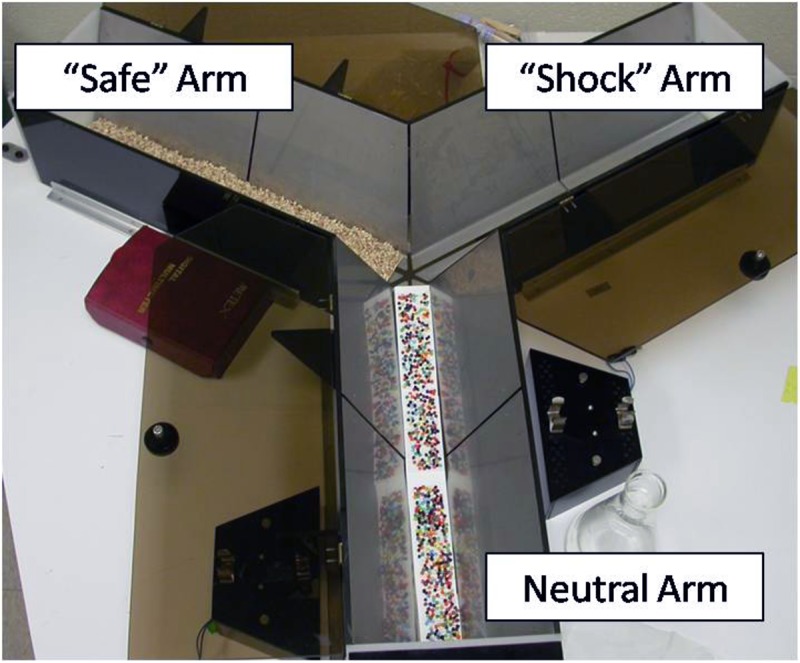
Picture of the Y-maze used for the Generalization Test in Experiment 2. The neutral arm served as the main stem of the maze. Flooring for the neutral arm consisted of a beaded cardboard insert; a novel environment and texture. The left and right arms of the maze were counterbalanced so there was an equally likely chance of the right arm resembling the “shock” or “safe” arm. The “safe” arm consisted of fresh corncob bedding normally used to line the bottom of the animal’s home cage. The “shock” arm had metal floors similar to those in the water-motivated inhibitory avoidance task in which animals were previously shocked.

In Experiment 2, a trough-shaped Y-maze constructed of stainless steel was used to examine the strength of the memory for the footshock experienced in Experiment 1 (**Figure 2**). The three alleys of the maze were each 49 cm long × 18.5 cm high. The floor and ceiling were 4 and 19 cm wide, respectively. The floor of the stem arm was covered by a removable cardboard panel embedded with tiny beads. This served as a neutral environment the animals had never experienced. The left and right alleys were constructed of two stainless steel plates separated lengthwise by a 0.5 cm gap, similar to the shock context in Experiment 1. However, an additional cardboard panel with fresh bedding was inserted into either the left or right arm in a counterbalanced fashion. This created two distinct arms; one that resembled a safe environment (fresh bedding normally used to line the rat’s homecage) and one that resembled the context in which the animals had been previously shocked (steel plates). In addition, a water spout was positioned at the end of each alley to resemble the initial training environment. The bedding was changed after each animal and the apparatus was cleaned with a 10% ethanol solution.

#### Generalization Testing

Forty-eight hours following footshock in the water-motivated inhibitory avoidance task of Experiment 1, a subset of animals were tested in the Y-Maze task. Specifically, only those animals that received either PBS or norepinephrine injections in the basolateral amygdala or hippocampus *and* PBS or muscimol 1 h later in the accumbens shell. Only the 1 h muscimol treatment groups were tested in the Generalization Test since there were no statistically significant differences between the 1 h and 7 h musicmol groups on any of the behavioral measures recorded in Experiment 1. Testing began by placing an animal in the neutral arm of the Y-maze. Each animal was allowed 300 s to explore the maze. The location of the “safe” and “shock” arms were counterbalanced to eliminate possible confounds that may evolve from left or right biases. Measurements included (1) latency to enter the “shock” arm, (2) latency to lick from the spout in the shock arm and (3) cumulative time spent drinking from the water spout.

#### Statistical Analysis

The behavioral measures from the Y-maze task are expressed as mean ± standard errors (SE). Between-group comparisons for the behaviors measured during retention testing were made with a 2 × 2 × 2 analysis of variance (ANOVA) followed by *post hoc* tests.

## Results for Experiment 1

### Basolateral Amygdala Injections

#### Phase 1 Retention Testing

##### First lick latency in the shock compartment

A 2 × 2 ANOVA on mean latency to first lick during Phase 1 of retention testing did not differ statistically between the separate control and treatment groups, *F*(2,30) = 1.27, *p* > 0.05. Subjects from each group displayed a lick response during Phase 1 and the mean latency for this response ranged from 25.3 to 51.2 s. The means for each group given basolateral injections followed 1 or 7 h later by infusions into the accumbens shell are: PBS/1 h PBS *M* = 29.32, PBS/1 h Muscimol *M* = 35.52, PBS/7 h Muscimol *M* = 39.86, NE/1 h PBS *M* = 39.39, NE/1 h Muscimol *M* = 25.36, and NE/7 h Muscimol *M* = 51.20).

##### Escape latency from the shock compartment

A 2 × 2 ANOVA on mean latency to exit the shock context and enter the white compartment during the 1-min retention test revealed no significant interaction between the treatment groups, *F*(2, 30) = 1.34, *p* > 0.05. All animals remained in the shock compartment without crossing into the illuminated area of the apparatus for a similar amount of time. The mean escape latency and number of rats per group that remained in the dark compartment for the 60 s. retention test are included in parenthesis. (PBS/1 h PBS 60.0 ± 0.0, *n* = 5; PBS/1 h Muscimol 60.0 ± 0.0, *n* = 5; PBS/7 h Muscimol 52.9 ± 15.7, *n* = 4; NE/1 h PBS 46.6 ± 23.9, *n* = 6; NE/1 h Muscimol 60.0 ± 0.0, *n* = 5; and NE/7 h Muscimol 56.4 ± 9.4, *n* = 4).

#### Phase 2 Retention Testing

##### Contextual memory

**FIGURE 3:**
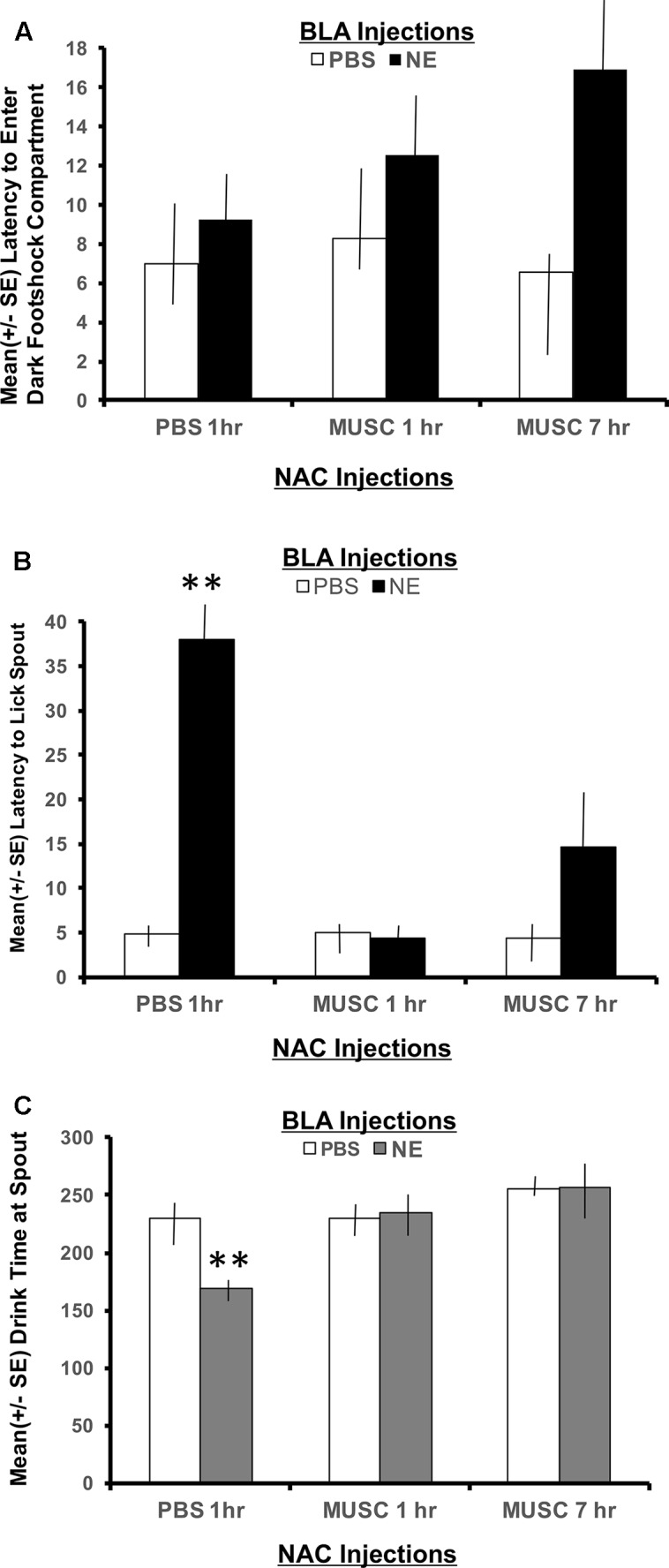
**(A)** Mean (+SE) latency for subjects with bilateral basolateral and accumbens shell cannulae implants to enter the compartment where shock was delivered 24 h earlier. There were no group differences in the time it took animals to enter the shock context. A 2 × 2 ANOVA revealed no significant differences between PBS controls and all other treatment groups to enter the dark, footshock compartment of the apparatus. **(B)** Mean (+SE) latency for animals with basolateral amygdala and accumbens shell cannulae implants to lick the spout after entering the shock compartment. Although all groups readily entered the dark compartment, only animals given norepinephrine (NE) in the basolateral amygdala (BLA) and PBS in the accumbens (NAC) took significantly longer to traverse the dark compartment and initiate licking from the spout (^∗∗^*p* < 0.01). The enhancement produced by BLA infusion of NE was blocked by intra-accumbens suppression of neuronal activity with Muscimol either 1 or 7 h following the NE injections. ^∗∗^ denotes *p* < 0.01. **(C)** Mean (+SE) time spent drinking from the water spout in the footshock compartment in animals with basolateral and accumbens shell cannulae implants. The cumulative time spent drinking was significantly reduced in animals given intra-basolateral infusion of norepinephrine and intra-accumbens PBS (*p* < 0.01). Inactivation of the accumbens shell with muscimol 1 or 7 h later blocked this effect. Animals in the NE/1 h MUSC and NE/7 h MUSC groups drank for a similar amount of time as non-shock control animals. ^∗∗^ denotes *p* < 0.01.

The second phase of retention testing began by placing each subject in the white compartment of the apparatus facing away from the retractable door. After a 30 s delay, access to the dark (shock) compartment was made possible by lowering a guillotine door that separates the two sections of the apparatus. Latency to enter the dark compartment was then recorded and served as an index of memory for the footshock experienced 24 h earlier. A 2 × 2 ANOVA indicated no significant interaction between basolateral amygdala (BLA) and accumbens treatments for the latency to enter the shock compartment, *F*(2,30) = 1.00, *p* > 0.05. As shown in **Figure 3A**, all experimental groups spent a similar length of time in the white compartment before entering the context where footshock was administered 24 h previously.

##### Latency to first lick

The footshock administered on day 7 of training was not delivered until the subject approached the spout and initiated its first lick to drink. As such, the representations of this event that may be encoded into memory include, (1) the context in which the footshock occurred and (2) the instrumental action emitted before delivery of the footshock (approaching the spout to drink). To assess memory for the second possible representation, the time required to drink from the spout after entering the shock context as well as the duration of time spent drinking was recorded. As shown in **Figure 3B**, a 2 × 2 ANOVA revealed a significant interaction between BLA and accumbens treatments, *F*(2,30) = 4.55, *p* < 0.05. Each of the treatment groups readily entered the dark shock context containing the water spout at the beginning of Phase 2 testing. However, subjects in the group given posttraining infusion of norepinephrine in the BLA and PBS 1 h later in the accumbens shell (NE/1 h PBS) took significantly more time to initiate drinking from the water spout relative to all other treatment groups (*p* < 0.01).

The memory enhancing actions of norepinephrine on BLA functioning was attenuated by suppressing accumbens neuronal activity with muscimol. The lick latencies of subjects given muscimol into the accumbens either 1 or 7 h following the norepinephrine treatment were indistinguishable from those of PBS controls (*p* > 0.05) and significantly shorter than subjects given the same dose of norepinephrine in the BLA (NE/1 h PBS compared to NE/1 h Muscimol, *p* < 0.01 and NE/7 h Muscimol, *p* < 0.01).

##### Time spent drinking

The duration of time spent consuming water from the spout after initial contact was made was also recorded. This measure served as an additional index of memory for the lick-footshock association developed after the Day 7 training. A 2 × 2 ANOVA revealed a significant interaction between treatment groups for the mean time spent drinking from the spout where footshock occurred 24 h earlier, *F*(2,30) = 4.60, *p* < 0.05 (**Figure 3C**). As assessed by *post hoc t*-tests, animals in the NE/1 h PBS group spent significantly less time drinking from the spout relative to all other treatment groups (*p* < 0.01 for PBS/1 h PBS, PBS/1 h Muscimol, PBS/7 h Muscimol, NE/1 h Muscimol and NE/7 h Muscimol). Again, regardless of the time delay between BLA and accumbens injections (1 h vs. 7 h), muscimol in the accumbens blocked the influence of activating noradrenergic receptors in the basolateral amygdala. Muscimol given 1 or 7 h later in PBS animals, however had no effect on performance as compared to PBS/1 h PBS animals (*p* > 0.05 for PBS/1 h Muscimol and PBS/7 h Muscimol).

### Hippocampus Injections

#### Phase 1 Retention Testing

##### First lick latency in the shock compartment

A 2 × 2 ANOVA on mean latency to first lick during Phase 1 of retention testing showed a significant difference between the treatment groups, *F*(2,28) = 3.66, *p* < 0.05. *Post hoc* comparisons revealed that the group given post training ventral hippocampal infusion of norepinephrine and accumbens PBS refrained from licking the spout significantly longer than all other groups during this first phase of testing (PBS/1 h PBS *M* = 35.88, PBS/1 h Muscimol *M* = 30.12, PBS/7 h Muscimol *M* = 52.83, NE/1 h PBS *M* = 60.00, NE/1 h Muscimol *M* = 37.36, and NE/7 h Muscimol *M* = 38.47). The remaining control and treatment groups displayed similar latencies to lick the spout during this initial test.

##### Latency to escape the shock compartment

Similar to the findings obtained with the basolateral amygdala groups, the mean latency to exit the shock context and enter the white compartment was not statistically different between the separate hippocampal treatment groups as assessed by a 2 × 2 ANOVA (HIPP × accumbens treatment), *F*(2,28) = 2.22 *p* > 0.05 (data not shown). All experimental groups remained in the shock compartment for a similar amount of time during this initial phase of retention testing. The mean escape latency and number of rats per group that remained in the dark compartment for the 60 s retention test are included in parenthesis. (PBS/1 h PBS 56.0 ± 8.9, *n* = 4; PBS/1 h Muscimol 60.0 ± 0.0, *n* = 5; PBS/7 h Muscimol 41.2 ± 21.4, *n* = 3; NE/1 h PBS 55.0 ± 11.2, *n* = 4; NE/1 h Muscimol 49.8 ± 15.2, *n* = 5; and NE/7 h Muscimol 54.7 ± 13.0, *n* = 4).

#### Phase 2 Retention Testing

##### Contextual memory

**FIGURE 4:**
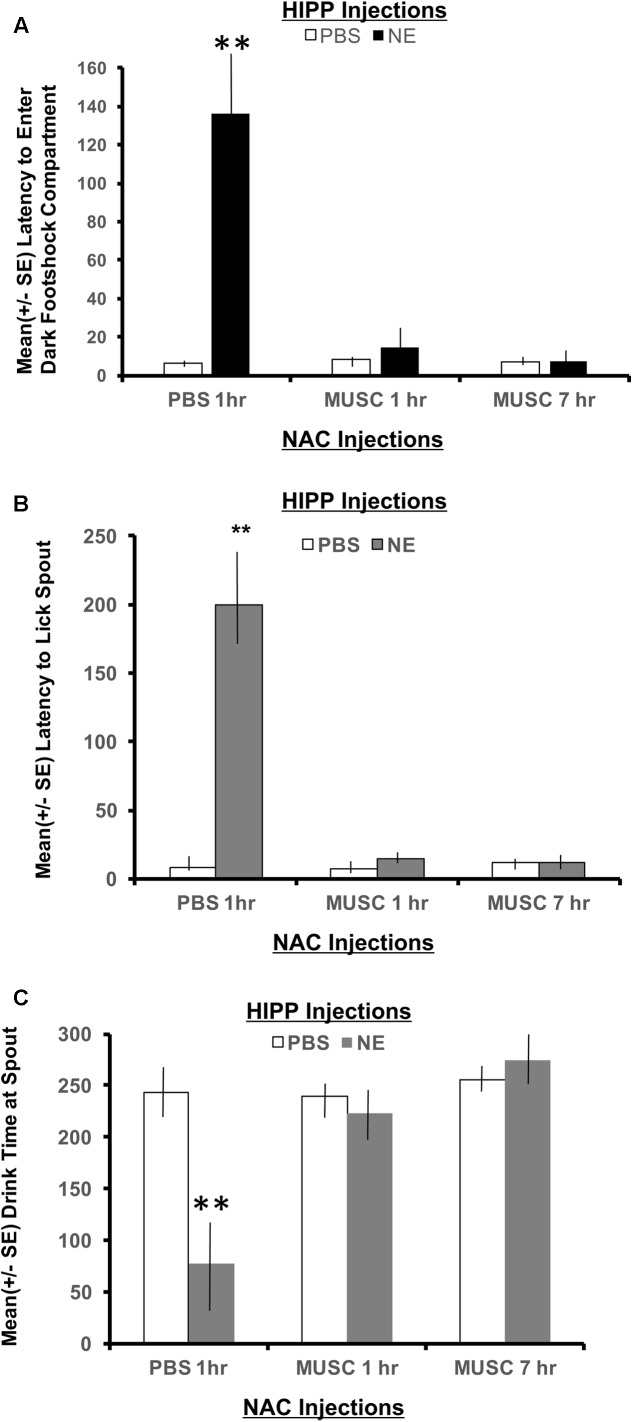
**(A)** Mean (+ SE) latency to enter the compartment where shock was administered 24 h previously in animals with ventral hippocampal and accumbens shell cannulae implants. Only animals given norepinephrine in the hippocampus took significantly longer to enter the context where footshock had been delivered 24 h previously (*p* < 0.01). Infusion of muscimol in the accumbens either 1 or 7 h later attenuated the memory enhancing effect (*p* < 0.01 for NE/1 h MUSC and NE/7 h MUSC groups compared to NE/PBS group). These findings are in direct contrast to those depicted in **Figure 3A** for animals given the same dose of norepinephrine in the basolateral amygdala. ^∗∗^ denotes *p* < 0.01. **(B)** Mean (+SE) latency for animals with ventral hippocampal and accumbens shell cannulae implants to initiate licking from the spout after entering the dark compartment. Not only did animals in the NE/1 h PBS group take longer to enter the shock context, but they also took significantly longer to initiate drinking from the spout compared to all other treatment groups (^∗∗^*p* < 0.01). **(C)** Mean (+SE) time spent drinking from the water spout located in the dark compartment on a retention test given 48 h after training with a footshock. Disruption of accumbens neuronal functioning via infusions of muscimol either 1 or 7 h later blocks the memory enhancement of activating ventral hippocampal neurons with norepinephrine. This effect is illustrated by the length of time these groups spent drinking compared to animals treated with the same dose of norepinephrine in the hippocampus and PBS in the accumbens. Only NE/1 h PBS animals spent a significantly less amount of time drinking from the water spout (^∗∗^*p* < 0.01).

To measure memory for the context where footshock was delivered, the latency to enter the shock compartment was measured from the beginning of Phase 2 (animals start in the white compartment). As shown in **Figure 4A**, a 2 × 2 ANOVA revealed a significant interaction between hippocampal (HIPP) and accumbens infusions, *F*(2,28) = 4.22, *p* < 0.05. All treatment groups preformed in a similar fashion, except the NE/1 h PBS group. Animals given norepinephrine in the hippocampus and PBS in the accumbens an hour later, took significantly longer to exit the white “safe” compartment and enter the dark context where footshock was delivered 24 h earlier (*p* < 0.01 compared to all treatment groups). Infusion of muscimol in the accumbens either 1 or 7 h later attenuated the memory enhancing effect of activating the hippocampus with norepinephrine (*p* < 0.01 for NE/1 h Muscimol and NE/7 h Muscimol groups compared to NE/1 h PBS group).

##### Latency to first lick

A 2 × 2 ANOVA also revealed significant differences between treatment groups on the latency to lick the spout after entering the dark compartment, *F*(2,28) = 10.80, *p* < 0.01. The NE/1 h PBS group took significantly longer to enter the shock context than any other group and also required a significantly longer period of time to lick the spout (*p* < 0.01, compared to all treatment groups; **Figure 4B**). Muscimol infusions into the accumbens shell that were delayed either 1 or 7 h posttraining blocked the effects of noradrenergic activation of the ventral hippocampus and attenuated the extended time to both enter the footshock compartment and initiate licking from the water spout.

##### Time spent drinking

There was also a significant interaction between treatment groups on the cumulative time spent drinking from the spout as revealed by a two-way ANOVA, *F*(2,28) = 8.54, *p* < 0.01 (**Figure 4C**). *Post hoc t*-tests revealed that animals in the NE/1 h PBS group spent significantly less time drinking from the spout relative to all other treatment groups (*p* < 0.01 for PBS/1 h PBS, PBS/1 h Muscimol, PBS/7 h Muscimol, NE/1 h Muscimol and NE/7 h Muscimol). Despite the time delay between HIPP and accumbens injections (1 h vs. 7 h), muscimol in the accumbens blocked the influence of activating noradrenergic receptors in the basolateral amygdala. Muscimol given 1 or 7 h later in PBS animals, however had no effect on performance as compared to PBS/1 h PBS animals (*p* > 0.05 for PBS/1 h Muscimol and PBS/7 h Muscimol).

### Comparison between BLA and HIPP Treatments

Since all groups in Experiment 1 experienced identical training and footshock procedures, it was possible to determine whether posttraining treatments rendered within the basolateral amygdala versus the hippocampus differentially affects memory for the separate responses that are measured during retention testing. The water-motivated inhibitory avoidance task has been used to assess the contribution of amygdala processing during arousing situations (Miyashita and Williams, 2002). The task design also has strong contextual features that require hippocampal processing. Thus, an assessment of performance in groups given PBS or NE within these structures and only PBS in the accumbens shell should determine which aspects of this task are more sensitive to amygdala versus hippocampal processing.

A 2×2 ANOVA (amygdala or hippocampus × norepinephrine or PBS) revealed significant differences between groups for two critical measures. Animals given PBS in either the basolateral amygdala or ventral hippocampus perform in a similar fashion across measures of latency to enter the shock compartment and latency to initiate licking from the spout after entering the dark compartment. However, there are significant differences in basolateral amygdala norepinephrine infusions compared with hippocampal norepinephrine infusions. Animals given norepinephrine in the hippocampus took significantly longer to enter the shock compartment, *F*(1,18) = 4.33, *p* < 0.05, and drink from the water spout, *F*(1,18) = 7.47, *p* < 0.05, relative to the basolateral groups given the same treatment (*data not shown*).

## Results for Experiment 2

A second experiment was conducted to assess the strength of the representation of footshock training retained in memory for the norepinephrine and muscimol treatment groups (both BLA and hippocampal) used in Experiment 1. For this purpose, only subjects given amygdala or hippocampal PBS or norepinephrine followed by accumbens treatment of 1 h PBS or 1 h muscimol during Experiment 1 were given a generalization test in a Y-maze apparatus. Two basolateral and two hippocampus animals were unable to complete this task and were excluded from data analysis. The Y-maze apparatus was modified such that only one of the maze alleys contained the same contextual attributes (i.e., metal walls and footshock plates) that were present during footshock delivery in Experiment 1 whereas the remaining two alleys were constructed with completely different contextual features.

### Basolateral Amygdala versus Hippocampus

**FIGURE 5:**
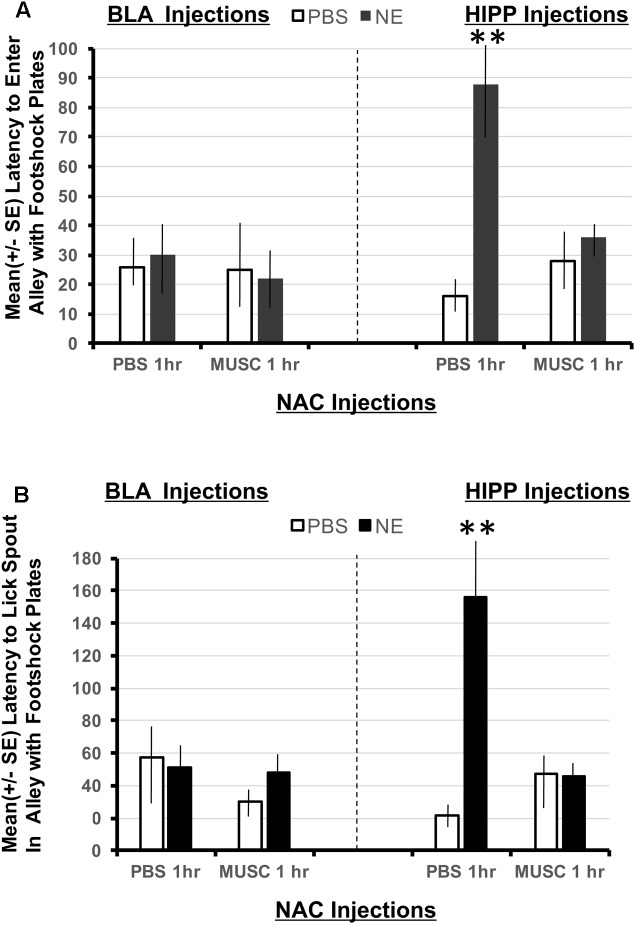
**(A)** Mean (+SE) latency to enter the Y-maze alley resembling the footshock context in animals with accumbens shell cannulae implants and implants in either the basolateral amygdala or ventral hippocampus. Only animals given norepinephrine (NE) in the hippocampus took significantly longer to enter the arm of the Y-maze that resembled the footshock context (*p* < 0.01). Although the shock and injection was given 48 h previously, these animals maintain high contextual memory for the shock compartment. This effect is blocked in animals given muscimol in the accumbens. ^∗∗^ denotes *p* < 0.01. **(B)** Mean (+SE) latency to lick from the water spout located in the Y-maze ally resembling the footshock context in animals with accumbens shell cannulae implants and implants in either the basolateral amygdala or ventral hippocampus. Animals in the BLA-NE/PBS not only readily entered the similar shock context, but they also readily drank from the spout located at the end of the arm. However, the same dose of norepinephrine in the hippocampus produced significantly longer latencies to lick from the spout compared to all other treatment groups (*p* < 0.01).

Using a 2 × 2 × 2 ANOVA (BLA/HIPPx PBS/NE x NAC PBS/NAC Muscimol 1 h), it is possible to determine if animals given amygdala or hippocampus treatments perform differently in this task designed to assess the strength of the footshock representation in Experiment 1. The ANOVAs revealed a significant interaction between amygdala, hippocampal and accumbens treatments on the latency to enter the shock resembling arm *F*(1,32) = 6.01, *p* < 0.05 as well as the latency to first lick from the spout in the arm that resembled the shock compartment, *F*(1,32) = 8.38, *p* < 0.01. As shown in **Figure 5A**, only animals with hippocampal infusions of norepinephrine and intra-accumbens PBS took significantly longer to enter the shock arm (*p* < 0.01 compared to all BLA and HIPP treatment groups). These animals also took significantly more time to initiate licking from the spout located in the “shock” arm (*p* < 0.01 compared to all BLA and HIPP treatment groups; **Figure 5B**). Disruption of accumbens processing 48 h previously with muscimol attenuated these two effects within hippocampus treated animals. Infusions of norepinephrine in the basolateral amygdala did not produce any appreciable differences in performance as compared to PBS/PBS controls.

## Discussion

Although findings from anatomical and physiological studies revealed that amygdala and hippocampal inputs converge on single neurons in the accumbens shell (French and Totterdell, 2003), evidence suggesting that accumbens processing is necessary to integrate new learning experiences from these limbic areas into memory is scarce. Moreover, experimental findings implicating the actual time frame in which the accumbens contributes to encoding novel events into memory storage has not been successfully documented. Results emerging from the present experiments are instrumental in addressing both of these shortcomings in the literature.

Findings from Experiment 1 confirm previous studies demonstrating that posttraining activation of noradrenergic receptors within the basolateral amygdala or hippocampus facilitate subsequent retention performance (Bevilaqua et al., 1997; Izumi and Zorumski, 1999; Birthelmer et al., 2003; Miranda et al., 2003; van Stegeren et al., 2005, 2008; Roozendaal et al., 2006, 2008; Tully et al., 2007; Dommett et al., 2008). The present results also extend these findings by revealing that noradrenergic activation of the amygdala or hippocampus differentially facilitates the category of representations formed after emotionally arousing events involving unexpected footshock. For example, noradrenergic activation of the amygdala facilitates memory for response specific representations directly associated with footshock delivery (**Figure 3B**), whereas activation of the hippocampus enhances memory for the context in which the emotionally arousing footshock is delivered (**Figure 4A**).

Second, animals given intra-hippocampal infusions of norepinephrine (HIPP-NE) took significantly longer than all other treatment groups to enter the Y-maze alley in Experiment 2 that was contextually similar to the dark compartment where footshock was delivered in the first study. This finding indicates that noradrenergic activation of the hippocampus not only leads to more stable representations of the footshock event over time, but this memory also generalizes to new learning conditions involving similar contextual stimuli. In contrast, the response specific memory associated with licking the spout that was evident in subjects given intra-amygdala infusions of norepinephrine was not as robust when this group was placed in the Y-maze although it contained similar contextual features. These animals readily entered the context of the Y-maze containing the metal footshock plates and did not hesitate before drinking from the water spout (**Figures 5A,B**).

The most intriguing findings of the current study reveal that the consequences of activating noradrenergic receptors in the amygdala or hippocampus are mediated in part by actions initiated within the accumbens shell. The data show that inactivation of the shell with the GABAergic agonist muscimol, attenuates memory enhancement produced by activating either the amygdala or hippocampus. This attenuation in memory was evident when neuronal activity in the accumbens shell as interrupted either 1 or 7 h after the limbic drug infusions. These results extend what is currently known regarding the time frame in which the accumbens contributes to mnemonic processing (Lorenzini et al., 1995) and demonstrates that this activity is critical during the initial and late stages of consolidation.

### Differences in Amygdalar and Hippocampal Processing

Results from electron microscopy studies confirm that both the amygdala and hippocampus converge on single output neurons in the accumbens shell (French and Totterdell, 2003). Based on this anatomical arrangement, accumbens neurons are in an ideal position to integrate representations of new learning experiences that are initially processed by the amygdala and hippocampus. In support of this view, Roozendaal et al. (2001) found that post-training infusions of compounds that facilitate retention when given in either the amygdala or hippocampus, were ineffective in influencing memory in animals given pre-training accumbens lesions. Other manipulations that interrupt normal accumbens synaptic activity such as microinfusions of tetrodotoxin also impair retention of a footshock given in a similar inhibitory avoidance task even when the injections are delayed for 90 min following training (Lorenzini et al., 1995). Together, these results suggest that processing in the hippocampus or amygdala alone may not be sufficient to influence memory, but may require additional integration within the accumbens.

Results from the current study are in concordance with previous findings (Bevilaqua et al., 1997) that noradrenergic activation of the amygdala or hippocampus facilitates memory for an arousing footshock experience. Using a one-trial step down inhibitory avoidance task, Bevilaqua et al. (1997) found that basolateral activation enhances memory only when norepinephrine is administered immediately post-training. Noradrenergic activation of the hippocampus, however, enhances memory for the footshock experience when administered 0, 3, or 6 h post-training. These data suggest that memory modulation in the hippocampus occurs for up to 6 h compared to amygdala modulation. However, measures of step-down latency used in the previous study fail to discern the specific contributions each limbic structure provides to the memory representation of the footshock experience. For example, information conveyed from the basolateral amygdala to the accumbens shell plays a crucial role in the learning and storing into memory the motivational value of stimuli. In contrast, hippocampal afferents to the accumbens provide information regarding contextual features of the environment. Results from the present work not only show that noradrenergic activation of limbic structures enhances memory, but reveal key differences in activating the amygdala or hippocampus.

The behavioral paradigm used in Experiment 1, was developed to dissociate representations in memory for the contextual versus the response specific aspects of learning that occur following unexpected footshock delivery. Therefore, it was possible to evaluate the differential contributions each limbic structure provides following activation. Results show that activation of the basolateral amygdala had no effect on the latency to enter the context where footshock had been administered 24 h previously (**Figure 3B**). However, this treatment was shown to facilitate memory on measures relating to response specific aspects of the task such as latency to lick the spout and the cumulative time spent drinking (**Figures 3B,C**). In contrast to amygdala activation, infusions of norepinephrine in the ventral hippocampus facilitate memory for the context in which the footshock transpired (**Figure 4A**). Because these animals took significantly longer to enter the shock context, they also have longer latencies to initiate drinking from the water spout. These behavioral findings provide functional evidence that supports electrophysiological data showing that hippocampal activation generates longer durations of accumbens activity as compared to amygdala stimulation (Grace, 2000). Together with the current behavioral data, it can be suggested that the longer periods of neuronal activity in the accumbens in response to hippocampal activation reflect the attention required to process contextual cues. During initial training in the water-motivated inhibitory avoidance task, animals learn that a context that was once pleasant (provided the availability of water) is now aversive (footshock). On the other hand, it can be suggested that the brief period of accumbens neuronal activity following amygdala stimulation reflects event-related processing. This is supported by findings that, although animals infused with norepinephrine in the amygdala readily enter the shock compartment, they still require a significantly longer period of time to begin drinking from the spout (last response emitted before the footshock was delivered). The difference in the magnitude of memory enhancement between basolateral and hippocampal animals shown in the current study provide behavioral support for the view that during emotionally salient events, hippocampal input may dominate with contextual processing compared to basolateral amygdala input, which may tag the affective value of the situation (Grace, 2000; Gill and Grace, 2011).

In contrast to the current results, a separate study found the basolateral amygdala to be involved in contextual learning and that processing in the hippocampus is not required 6 h post-training (Sacchetti et al., 1999). Several procedural dissimilarities underlie the discrepancy reported between these data and the current findings. First, it should be noted that Sacchetti et al. (1999) used Pavlovian fear conditioning procedures such that a tone preceded a footshock. The pairing of tone with a shock occurred seven times, giving the animal ample time to associate the tone and shock with the context. In the current study, animals were given only a single footshock that did not persist beyond 2 s before they escaped into the white compartment. An additional difference between the two behavioral paradigms is that the current study required an instrumental response of approaching the spout before the footshock was delivered. This allows for a more precise association between action and stimulus (shock). A third discrepancy that should be noted is the target area of the hippocampus. Sacchetti et al. (1999) found that processing in the dorsal hippocampus is not required 6 h after the footshock experience. This means that 6 h post-training, blockade of dorsal hippocampal neurons has no influence on memory. But the current study investigated the contribution of ventral hippocampal processing to memory consolidation. If the ventral hippocampus were similar to the dorsal, then only animals given norepinephrine in the hippocampus and muscimol in the accumbens1 h later would show attenuation in contextual memory. However, the current results showed that an intact pathway from ventral hippocampus to accumbens is required 7 h post-training in order to facilitate memory for where the footshock occurred. Taken together, these findings suggest that dorsal and ventral hippocampal areas not only differ in the pattern of innervation to the accumbens, but also in the length of time that neuronal processing is required to facilitate contextual and spatial memories.

### Gating of Limbic Information within the Nucleus Accumbens Shell

Several studies indicate that accumbens neurons require activation from more than one source to reach threshold (DeFrance et al., 1985; Callaway et al., 1991). This constraint on activity may explain why accumbens neurons receive inputs from several memory related areas. In particular, projections from the basolateral amygdala and ventral hippocampus converge monosynaptically on projection neurons within the caudomedial region of the accumbens shell (French and Totterdell, 2003; Britt et al., 2012; Correia et al., 2016). Because the accumbens receives converging inputs from multiple areas, it is important to understand how separate inputs may regulate neuronal firing in this structure. An emerging idea in the literature dealing with the functionality of the nucleus accumbens is the hypothesis of neural networks. Pennartz et al. (1994) propose that the accumbens is comprised of neuronal ensembles. These ensembles are best understood in terms of their collective activation.

McGinty and Grace (2009) demonstrated that nucleus accumbens neurons integrate limbic and cortical innervations depending on the intensity and timing of inputs. Specifically, weak stimulation of two inputs generates more excitation of accumbens neurons than activation of either structure alone. When these stimulations occur at the same time, accumbens neurons become active. This electrophysiological characteristic of neurons in the accumbens establishes a coincidence detection system such that areas that fire together have direct influence over accumbens activity. However, an interesting finding by McGinty and Grace (2009) showed that strong activation of one input may disrupt processing of the second input. This electrophysiological feature may serve as the mechanism underlying behavioral differences in hippocampal and amygdala activation reported in the present study. Although activation of both limbic structures facilitates memory for certain aspects of the water-motivated inhibitory avoidance task, the magnitude of the facilitation was greater in subjects receiving post-training noradrenergic activation of the ventral hippocampus. The reason may be due to the fact that delivery of the footshock in Experiment 1 continued the whole length of the dark compartment until animals escaped into the safe/white compartment. The animals remained in the white compartment for 30 s before the retractable door was raised. During this 30 s period of time, animals could see the dark compartment and form a distinct representation between the “dark” shock and “illuminated” safe compartments of the apparatus. This component of the training procedure may account for the stronger degree of activation in the hippocampus. This strong activity may have disrupted amygdala processing as proposed by McGinty and Grace (2009), leading to a facilitation in contextual measures in animals with norepinephrine infusions in the hippocampus as compared to the amygdala.

### Significance of Accumbens Involvement in Long-Term Consolidation

The accumbens not only plays a role in the integration of information emanating from the hippocampus or amygdala, but is also involved in the consolidation of these processes into memory. For example, rats with accumbens lesions fail to modify response latencies or reaction time to cues that lead to aversive outcomes, such as the delivery of quinine in the place of an anticipated liquid sucrose reward (Schoenbaum and Setlow, 2003). The consolidation of memory for these and other types of emotionally arousing events is a time dependent process that does not happen instantly, but rather occurs over hours. Studies employing functional inactivation techniques to produce reversible lesions demonstrate that neuronal activity in the accumbens is crucial for consolidation for at least 90 min following learning (Lorenzini et al., 1995).

While findings from the current study are in agreement with previous results (Lorenzini et al., 1995), they also extend what is currently known about the integrative nature of accumbens neurons and the timeframe in which accumbens processing is required. First, the present work demonstrates that activation of the amygdala or hippocampus is not sufficient to enhance memory when accumbens activity is disrupted with muscimol. Second, results from the present study determine the temporal window in which neurons from the accumbens are required to process the beneficial information emanating from the amygdala or hippocampus. Findings show that without accumbens processing 1 or 7 h post-training, memory for a footshock experience is attenuated despite limbic activation. This timeframe corresponds to phases of synaptic plasticity documented in the hippocampus up to 6 h post-training with microarray analysis (O’Sullivan et al., 2007).

## Conclusion

This study provides evidence that blocking accumbens functioning with muscimol an hour or even 7 h following amygdala or hippocampus activation attenuates the improvement in memory seen following noradrenergic activation of the amygdala or hippocampus alone. These findings suggest that the accumbens shell plays an integral role modulating information initially processed by limbic structures following exposure to emotionally arousing events. Additionally, results are integral in determining the involvement of the accumbens shell in long-term consolidation processes lasting over 6 h.

## Author Contributions

The experiments were designed in collaboration between EK and CW. EK conducted the studies and analyzed the data. The final manuscript was written together by both authors.

## Conflict of Interest Statement

The authors declare that the research was conducted in the absence of any commercial or financial relationships that could be construed as a potential conflict of interest.
